# Serum miR-34a-5p and miR-199a-3p as new biomarkers of neonatal sepsis

**DOI:** 10.1371/journal.pone.0262339

**Published:** 2022-01-06

**Authors:** Omayma O. Abdelaleem, Shereen Rashad Mohammed, Hassan S. El Sayed, Sherin Khamis Hussein, Doaa Y. Ali, Mostafa Y. Abdelwahed, Sylvana N. Gaber, Nada F. Hemeda, Rehab G. Abd El-Hmid

**Affiliations:** 1 Faculty of Medicine, Departments of Medical Biochemistry and Molecular Biology, Fayoum University, Fayoum, Egypt; 2 Faculty of Medicine, Departments of Pediatrics, Fayoum University, Fayoum, Egypt; 3 Faculty of Medicine, Departments of Clinical Pathology, Fayoum University, Fayoum, Egypt; 4 Faculty of Medicine, Departments of Physiology, Fayoum University, Fayoum, Egypt; 5 Faculty of Medicine, Departments of Microbiology and Immunology, Fayoum University, Fayoum, Egypt; 6 Faculty of Agriculture, Department of Genetics, Fayoum University, Fayoum, Egypt; Nitte University, INDIA

## Abstract

**Background:**

Neonatal sepsis is a serious condition. Recent clinical studies have indicated that microRNAs (miRNAs) are key players in the pathogenesis of sepsis, which could be used as biomarkers for this condition.

**Patients and methods:**

A total of 90 neonates with sepsis and 90 healthy neonates were enrolled in this study. qRT-PCR was performed to measure the expression levels of serum miR-34a-5p and miR-199a-3p.

**Results:**

miR-34a-5p and miR-199a-3p serum levels were significantly reduced in neonates with sepsis compared with those in healthy neonates (P = 0.006 and P = 0.001, respectively). Significant correlations of miR-34a-5p and miR-199a-3p with each of TLC, RDW, RBS, and C-reactive protein (CRP) as well as SNAPII were observed, indicating their associations with the severity of neonatal sepsis.

**Conclusion:**

miR-34a-5p and miR-199a-3p may be useful as novel biomarkers in neonatal sepsis and may provide a new direction for its treatment.

## Introduction

Neonatal sepsis is a dangerous condition associated with elevated morbidity and mortality that is caused by infection of the blood in infants within 28 days of age. In sepsis, a systemic inflammatory response occurs due to the presence of infective pathogens, resulting in superimposed secondary infections and multiple organ failure [1].

Numerous biomarkers for the diagnosis and detection of sepsis at an early stage have been identified, such as C-reactive protein (CRP), procalcitonin (PCT), and interleukins (ILs), but they have poor specificity [2]. Blood culture is widely used in the diagnosis of sepsis, but the results of culture may be delayed and false negative results are often obtained [3–5]. Therefore, there is an urgent need to identify novel biomarkers for discriminating sepsis at an early stage in addition to the possibility of their use as therapeutic targets.

MicroRNAs (miRNAs) are short noncoding RNAs associated with the regulation of gene expression. miRNAs can target numerous protein-coding genes, playing roles in inflammation, immunity, apoptosis, and cell differentiation [6]. In addition, researches have shown that the expression of miRNA is modulated at an early stage of sepsis and this is positively correlated with severity and disease progression and are considered powerful endogenous factors regulating the inflammatory signaling cascade during sepsis. Also, blockage of their proinflammatory effects can effectively improve sepsis-related organ injury, offering new insights to detect possible biomarkers and therapeutic options for sepsis [7].

Recent studies reported that serum miRNAs can act as early biomarkers in many diseases, including sepsis [8,9]. Furthermore, miRNAs are considered perfect biomarkers for sepsis due to their selectivity, specificity, and stability [10].

miR-34a-5p and miR-199a-3p are miRNAs with vital roles in the development of inflammatory and autoimmune diseases [11–14], and they were proved to be involved in the regulation of TLR signaling and NF-κB-mediated inflammatory response which have main role in the process of sepsis [15]. However, the role of miR-34a-5p and miR-199a-3p in neonatal sepsis has yet to be studied.

The purpose of the current study is to evaluate the expression levels of miR-34a-5p and miR-199a-3p in neonates with sepsis and to evaluate their relationships with laboratory and clinical data in such patients.

### Patients and methods

The current study was performed on 90 neonates (33 males and 57 females), weighing 2.6±0.4kg, with clinical manifestations of sepsis. The subjects were separated into two groups as follows: (1) a sepsis group with positive blood culture of septicemia, which included 76 neonates; and (2) a sepsis group with negative blood culture, which included 14 neonates. Furthermore, 90 healthy control neonates (29 males and 61 females), weighing 2.7± 0. 7kg, were enrolled in this study.

The included newborns with sepsis were hospitalized in the neonatal intensive care unit, while healthy neonates were recruited from outpatients of the pediatrics Department, Fayoum University Hospital, during the period from December 2019 to September 2020. The cases of neonatal sepsis were diagnosed according to the criteria defined at the 2003 Kunming Neonatal Sepsis Definitions Conference [16].

Neonates with chromosomal anomalies, those with perinatal asphyxia, and those with intrauterine growth retardation were excluded from this study. Moreover, neonates whose mothers were diabetic, hypertensive or had any inflammatory or autoimmune diseases were excluded from the study. Written informed consent for each neonate was given by the parents or legal guardians. The present work was approved by the Ethics Committee of Fayoum University, in line with the Declaration of Helsinki. The Score for Neonatal Acute Physiology (SNAP II) was used in this study as a predictor of mortality and severity using six vital signs and laboratory test results [17].

### Sample preparation

Blood samples (3 ml of venous blood) at the moment of drawing the blood culture were collected in tubes containing separating gel and left to clot for 15 min at room temperature, followed by centrifugation at 3000 rpm for 10 min. The separated sera were stored at −80°C until RNA extraction.

### Extraction of miRNAs

Total RNA extraction, including of miRNAs, from serum samples was carried out using the miRNeasy Serum/Plasma kit (Qiagen, Valencia, CA, USA), in accordance with the manufacturer’s instructions. The extracted RNAs were subjected to RNA quantitation and purity measurement using a NanoDrop® (ND)-1000 spectrophotometer (NanoDrop Technologies, Inc., Wilmington, USA).

### Reverse transcription (RT) for synthesis of cDNA

Reverse transcription was performed on total RNA in reactions at RT with a final volume of 10 μl using the miRCURY LNA RT Kit (catalog no. 339340) (Qiagen, MD, USA).

### Quantitative real-time PCR (qPCR) for detection of miR-34a-5p and miR-199a-3p

Real-time PCR was carried out in the real-time cycle (PikoReal 24™ Real-Time PCR System; Thermo Scientific, Finland) using miRCURYLNA SYBR® Green Master Mix (Qiagen, MD, USA) and miRCURY LNA miRNA PCR Assays (Qiagen, MD, USA). Each reaction contained 5 μl of mercury LNASYBRGreen Master Mix, 1 μl of each primer, 3 μl of cDNA(diluted 1:30), and 1 μl of RNase-free water, reaching a final volume of 10 μl.

PCR cycling conditions were as follows: initial heat activation at 95°C for 2 min, followed by 40 cycles of denaturation at 95°C for 10 s and combined annealing/extension at 56°C for 60 s. Melting curve analysis was performed at 60°C–95°C to assess the specificity of the amplified products.

Serum expression levels of the studied miR-34a-5p and miR-199a-3p were estimated using miR-16-5p as an internal control, as it was the most stably expressed normalization candidate in our samples [18,19]. Previously established primers for miR-34a-5p, miR-199a-3p, and miR-16-5p were used (miR-34a-5p:catalog no. YP00204486 and lot number 201902080015–3; miR-199a-3p catalog no.YP00204536 and lot number 201910300008–3; miR-16-5p catalog no. YP00205702 and lot number 201910040131–3).

Gene expression relative to the internal control (2^−ΔCt^) was calculated. Fold change(FC) was considered using 2^−ΔΔCt^ for relative. If the FC is above 1, this means that the miRNA is overexpressed, while if it is below 1, this means that the miRNA is under expressed. The control value was set to 1 because–ΔΔCt for controls equals zero and 2^0^equals one [20].

### Organism isolation& identification

Each patient underwent blood culture testing using the BACTEC blood culture system 9050 (Becton Dickinson Diagnostic Instrument Systems, Sparks, MD, USA). From each presumptive positive vial, Gram staining and subculture on blood agar and MacConkey agar plates (Oxoid Ltd., UK) were performed. The isolates were identified after incubation at 37°C by Gram staining and analysis of colony characteristics. If budding yeast cells were seen in the Gram stain, subculture was performed in Sabouraud Dextrose Agar(Oxoid Ltd., UK) at room temperature. Germ tube was performed to differentiate *Candida albicans* from other *Candida* species. Antimicrobial sensitivity testing was performed for each bacterial isolate by the Kirby–Bauer disc diffusion technique. The results were clarified in accordance with Clinical Laboratory Standards Institute (CLSI) guidelines [21]. *Staph*.*aureus* ATCC 25923and *Escherichia coli* ATCC 25922 were used as controls to confirm the quality and accuracy of the testing procedures.

### Statistical methods

The current data were prepared and statistically analyzed using Statistical Package for Social Sciences version 18. The median, interquartile range (IQR), mean, and standard deviation (SD) were used for quantitative data. Chi-squared test was carried out for qualitative data, which were presented as number and percentages. Spearman’s correlation was performed to assess the correlations of miR-34a-5p and miR-199a-3p with clinical and laboratory data. A receiver operating characteristic (ROC) curve was created to identify the cut-off point, sensitivity, and specificity of miR-34a-5p and miR-199a-3p as biomarkers for neonatal sepsis. Differences were considered to be significant at P≤0.05. Adjusted P-values for multiple comparisons for each marker were calculated by using the Bonferroni correction method to account for the problem of multiple testing. P-value of 0.05 was divided by the number of comparisons i.e. number of clinical variables (0.05/9).

Sample size was calculated using (G power version 3). Minimal sample size of patients was 86 in each group needed to get power level 0.90, alpha level 0.05 and medium effect size of 0.5inthe studied biomarkers between the two groups.

## Results

### Characteristics of neonatal sepsis cases and healthy neonates

In this study, 90 neonates with sepsis as well as 90 healthy neonates were enrolled. The detailed demographic and clinical data are presented in Table 1. No statistically significant differences were observed between the case and control groups regarding age, sex, birth weight, or weight. However, significant differences were noted between the neonatal sepsis and control neonate groups concerning gestational age, maturity, Apgar score at 1 min, respiratory rate, heart rate, head circumference, and height.

**Table 1:** Demographic and clinical data of neonates with sepsis and control.

	Cases (N = 90)	Control (N = 90)	P-value
**Age/days**	11 (3−21)	7.1 (4−12)	0.711
**Sex:**			
**Male, n (%)**	33 (36.7)	29 (34.4)	0.530
**Female,n (%)**	57 (63.3)	61 (65.6)	
**Gestational age/weeks**	36.1± 1.9	37.5±0.6	<0.0001*
**Birth weight /kg**	2.5± 0.4	2.6± 0.7	0.113
**Apgar score at 1 min**	8.7±1.4	9.4±0.6	<0.0001*
**Respiratory rate**	66.2± 15.2	43.5± 3.9	<0.0001*
**Heart rate**	135.8± 17.2	121.3± 10.9	<0.0001*
**Head circumference**	33.5± 1.7	34± 0.9	0.009*
**Weight/kg**	2.6± 0.4	2.7± 0.7	0.243
**Height**	47.7± 3.6	49.7± 1.3	<0.0001*
**Maturity, n (%)**			
**Preterm**	39 (43.3)	10 (11.1)	<0.0001*
**Full term**	51 (56.7)	80 (88.9)	
**Sepsis onset**			
**Early onset; n (%)**	53 (58.8)		
**Late onset; n (%)**	37 (41.1)		
**PROM**			
**Yes, n (%)**	29 (32.2)		
**No, n (%)**	61 (67.8)		
**Convulsion**			
**Yes**	18 (20.0)		
**No**	72 (80.0)		
**Apnea**			
**Yes**	8 (8.9)		
**No**	82 (91.1)		
**Respiratory distress (RD)**			
**No**	19 (21.1)		
**RD1**	35 (38.9)		
**RD2**	28 (31.1)		
**RD3**	4 (4.4)		
**RD4**	4 (4.4)		

Data are expressed as median (IQR), mean ± SD, or n (%).

PROM: Premature rupture of membrane, RD 1: (mild distress):- tachypnea, workingalanasi.

RD2: (moderate distress):- subcostal and intercostal) due to moderate hypoxemia, RD3: (severe distress):- Grunting which is due to severe hypoxemia, RD4: Cyanosis and disturbed consciousness.*Significant at P <0.05.

Regarding the laboratory data as presented in Table 2, there were significant differences between neonatal cases and controls regarding TLC, platelets count, RDW, absolute lymphocyte count, I/T neutrophils, random blood sugar (RBS), CRP, pH, and HCO_3_.

**Table 2:** Laboratory data of neonates with sepsis and control group.

	Cases (N = 90)	Control (N = 90)	P-value
**TLC(×10⁹per L)**	17.72± 7.82	10.47± 2.98	<0.0001*
**Platelets(×10⁹per L)**	209.08± 185.52	335.46± 58.11	<0.0001*
**RDW**	16± 1.3	14.1± 0.6	<0.0001*
**Lymphocyte count (10** ^**3**^ **/μl)**	6.8± 4.7	3.7± 1.01	<0.0001*
**I/T neutrophils**	0.26± (0.11)	0.12± (0.05)	<0.0001*
**RBS(mg/dl)**	111±17.4	98.8±11.8	<0.0001*
**CRP(mg/dl)**	3.75±1.2	1.5±0.3	<0.0001*
**pH**	7.32±0.57	7.38±0.04	<0.0001*
**CO**_**2**_	41.2±11.1	41.9±4.5	0.550
**HCO**_**3**_	18.4±1.4	19.9±2	<0.0001*
**Mean_ABP(mmHg)**	53.7± 13.1		
**Urine output(ml/kg/h)**	1.2± 0.7		
**PO**_**2**_ **(mmHg)/FiO**_**2**_	2.2±0.7		
**SNAPII**	15 (5−26)		
**Culture,n (%)**			
Positive	76 (84.4)		
Negative	14 (15.6)		
**Organisms,n (%)**			
*Acinetobacterbaumannii*	5 (5.6)		
*Candida* species	10(11.1)		
*Enterobacter* species	9(10)		
*Enterococcus*	5(5.6)		
*Klebsiella* species	28(31.1)		
Negative	14(15.6)		
*Staph*.*aureus*	19(21.1)		
**Antibiotic resistance n (%)**			
AmpC beta lactamase	24(26.7)		
MDRO	13(14.4)		
MRSA	14(15.6)		
ESBL	5(5.6)		

Data are expressed as median (IQR), mean ± SD, or n (%).

TLC: Total leucocyte count, RDW: Red cell distribution width, I/T: Immature/total, RBS: Random blood sugar, CRP: C-reactive protein,ABP: Arterial blood pressure, SNAP: Score of neonatal acute physiology, MDRO: Multidrug-resistant organisms, MRSA: Methicillin-resistant *Staphylococcus aureus*, ESBL: Extended-spectrum beta-lactamases.

*Significant at P <0.05.

SNAPII was evaluated in neonates with sepsis to have a median (IQR) value of 15 (5–26). Furthermore, the results of blood culture in the sepsis group indicated that 76(84.4%) of the cases were blood culture-positive, while 14 (15.6%) were negative. The distribution of identified microorganisms in positive blood cultures showed that the Gram-negative bacteria represented 55% of the total, including *Klebsiella* species (36.8%), *Enterobacter* species (11.8%), and *Acinetobacter* species (6.5%). Overall, 32%of the positive blood culture results were Gram-positive organisms, mainly *Staph*.*aureus* (25%), followed by *Enterococcus* species (6.5%). Meanwhile, *Candida* species represented 13%.Overall, the results on antibiotic resistance among the positive blood cultures were as follows: 24(26.7%) for AmpC beta lactamase,14(15.6%) for methicillin-resistant *Staphylococcus aureus*(MRSA), 13(14.4%) involving multidrug-resistant organisms(MDRO), and 5(5.6%) for extended-spectrum beta-lactamases (ESBL) (Table 2).

### miR-34a-5p and miR-199a-3p serum levels in each group

**Fig 1:**
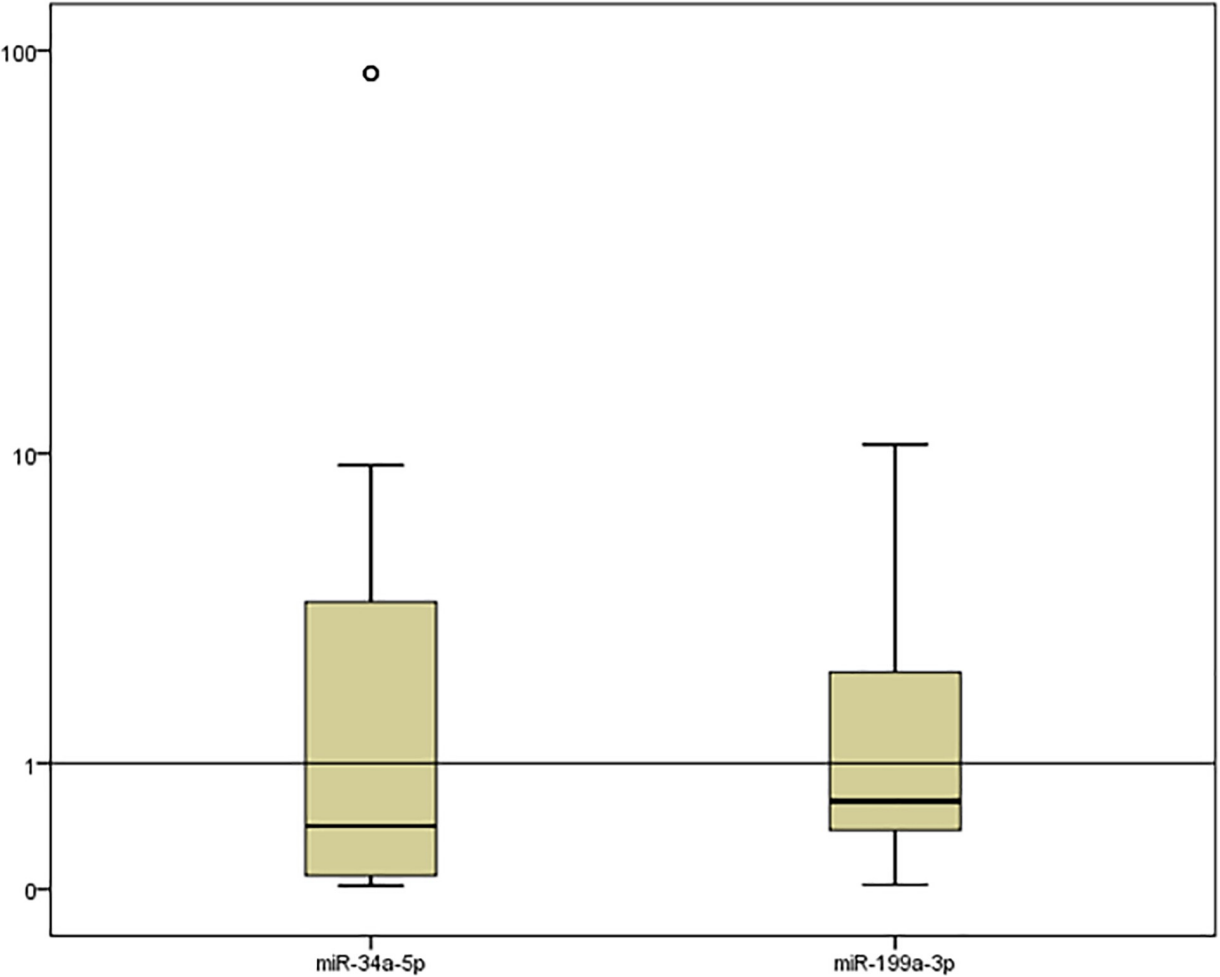
Box plot representation demonstrating the serum expression of miR-34a-5p and miR-199a-3p (fold change) in neonatal sepsis compared with the levels in healthy neonates. Data are presented as a box plot (median, upper, and lower quartiles). The horizontal line represents the expression levels of miR-34a-5p and miR-199a-3p in healthy neonates.

miR-34a-5p and miR-199a-3p FC serum levels were significantly low in neonates with sepsis compared with those in healthy neonates [median (IQR)FC of miR-34a-5p was 0.42 (0.08–3.86) with a P value of 0.006, while median (IQR)FC of miR-199a-3p was 0.62(0.38–2.41) with a P value of 0.001] Fig 1 and S1 Table.

### Associations of serum levels of miR-34a-5p and miR-199a-3p with clinical characteristics of patients with neonatal sepsis

To estimate the influence of the clinical characteristics and results of blood culture of neonatal sepsis cases on the levels of miR-34a-5p and miR-199a-3p, we analyzed the expression levels of these miRNAs according to the clinical characteristics and blood culture results in patients with neonatal sepsis after applying multiple testing correction (Table 3). The results showed that miR-34a-5p and miR-199a-3p were significantly downregulated in neonates with positive blood culture, mainly *Enterococcus* (P<0.0001, each). In addition, miR-34a-5p was demonstrated to be significantly downregulated with bacteria showing gram negative stain (P<0.0001). While, miR-199a-3p was verified to be significantly downregulated with bacteria showing gram positive stain (P<0.0001).

**Table 3:** Relationships of miR-34a-5p and miR-199a-3p with clinical data in neonates with sepsis.

	miR-34a-5p	P-value	miR-199a-3p	P-value
**Sex**				
Male (n = 33)	0.35 (0.19−3.86)	0.076	0.7 (0.51−2.41)	0.040
Female (n = 57)	0.48 (0.06−7.21)		0.57(0.21−4.89)	
**Gestational age**				
Preterm	0.33 (0.04−3.50)	0.024	0.62 (0.38−1.30)	0.050
Full term	0.51 (0.19−7.21)		0.63 (0.51−8.51)	
**PROM**				
Yes (n = 29)	2.38 (0.06−3.86)	0.962	2.41 (0.38−4.89)	0.839
No (n = 61)	0.33 (0.15−1.01)		0.62 (0.41−1.04	
**Convulsion**				
Yes(n = 18)	0.2 (0.04−88.03)	0.106	0.27 (0.02−1.3)	0.001
No(n = 72)	0.48 (0.17−3.86)		0.63 (0.41−4.89)	
**Respiratory distress (RD)**				
None(n = 19)	0.51 (0.48−8.88)	0.386	1.04 (0.21−10.48)	0.642
RD1(n = 35)	1.01 (0.04−7.21)		0.63 (0.57−8.51)	
RD2(n = 28)	0.27 (0.15−3.86)		0.51 (0.38−1.3)	
RD3(n = 4)	0.08 (0.08−0.15)		0.7 (0.63−0.7)	
RD4(n = 4)	0.22 (0.15−0.22)		0.62 (0.6−0.66)	
**Apnea**				
Yes(n = 8)	0.12 (0.02−0.22)	0.001	0.33 (0.03−0.62)	0.027
No(n = 82)	0.48 (0.15−3.86)		0.63 (0.41−2.41)	
**Sepsis onset**				
Early onset	0.35 (0.08−3.50)	0.307	0.62 (0.41−1.30)	0.912
Late onset	0.51 (0.19−3.86)		0.63 (0.38−2.30)	
**Culture**				
Positive (n = 76)	0.33 (0.07−2.94)	<0.0001*	0.57 (0.38−0.87)	<0.0001*
Negative (n = 14)	2.38 (1.01−88.03)		8.51 (1.3−8.51	
**Organisms**				
*Acinetobacterbaumannii* (n = 5)	0.51 (0.51−0.51)	<0.0001*	0.21 (0.21−0.21)	<0.0001*
*Candida* species (n = 10)	5.85 (0.06−9.32		0.57 (0.38−0.57	
*Enterobacter* species(n = 9)	7.21 (0.22-7.21		4.89 (0.62−4.89	
*Enterococcus* species(n = 5)	0.04 (0.04–0.04)		0.02 (0.02−0.02)	
*Klebsiella* species(n = 28)	0.12 (0.04−0.48)		0.6 (0.16−1.04)	
*Staph*.*aureus*(n = 19)	0.33 (0.19−8.88)		0.63 (0.41−10.48)	
**Gram stain type**				
Negative	0.15 (0.04−3.5)	<0.0001*	0.7 (0.38−2.3)	<0.0001*
Positive	0.27 (0.19−0.35)		0.54 (0.41−0.63)	
**Antibiotic resistance**				
AmpC beta lactamase (n = 24)	0.48 (0.07−3.86)	0.001	1.04 (0.48−2.41)	0.203
MDRO (n = 13)	0.08 (0.04−0.15)		0.57 (0.16−0.62)	
MRSA (n = 14)	0.35 (0.19−8.88)		0.57 (0.51−10.48)	
ESBL (n = 5)	0.33 (0.33−0.33)		0.63 (0.41−0.63)	

PROM: Premature rupture of membrane, MDRO: Multidrug-resistant organisms, MRSA: Methicillin-resistant *Staphylococcus aureus*, ESBL: Extended-spectrum beta-lactamases.

*Significant at P<0.006.

### Correlations of serum levels of miR-34a-5p and miR-199a-3p with clinical and laboratory parameters in sepsis group

Spearman’s analysis was used to evaluate the correlations of the serum levels of miR-34a-5p and miR-199a-3p with each of the clinical and laboratory variables (Table 4). Positive correlations were demonstrated between the serum level of miR-34a-5p and each of birth weight, weight, height, and platelet count, while it had negative correlations with TLC, RDW, RBS, CRP, as well as SNAPII. Regarding miR-199a-3p, it was found to be significantly positively correlated with birth weight, Apgar score at 1 min, weight, and platelet count, while it was negatively correlated with each of respiratory rate, heart rate, TLC, RDW, RBS,CRP, as well as SNAPII in neonates with sepsis.

**Table 4:** Correlations of serum levels of miR-34a-5p and miR-199a-3p with clinical and laboratory data in neonates with sepsis.

	miR-34a-5p r (P-value)	miR-199a-3p r (P-value)
**Age**	–0.048 (0.656)	–0.123 (0.246)
**Gestational age**	0.175 (0.098)	0.068 (0.522)
**Birth weight**	0.550 (<0.0001*)	0.332 (0.001*)
**Apgar score at 1 min**	–0.087 (0.417)	0.230 (0.029*)
**Respiratory rate**	–0.129 (0.227)	–0.211 (0.046*)
**Heart rate**	–0.147 (0.166)	–0.271 (0.010*)
**Head circumference**	0.205 (0.053)	0.181 (0.087)
**Weight**	0.342 (0.001*)	0.446 (<0.0001*)
**Height**	0.344 (0.001*)	0.196 (0.064)
**TLC**	–0.402 (<0.0001*)	–0.307 (0.003*)
**Platelets**	0.498 (<0.0001*)	0.508 (<0.0001*)
**RDW**	–0.309 (0.003*)	–0.237 (0.025*)
**Lymph**	–0.097 (0.362)	0.040 (0.707)
**I/T neutrophils**	0.116 (0.277)	0.059 (0.578)
**RBS**	–0.386 (<0.0001*)	–0.291 (0.005*)
**CRP**	–0.348 (0.001*)	–0.422(<0.0001*)
**pH**	–0.156 (0.142)	0.168 (0.113)
**CO**_**2**_	0.042 (0.694)	–0.007 (0.951)
**HCO**_**3**_	–0.150 (0.157)	–0.073 (0.494)
**Mean ABP**	0.012 (0.907)	0.107 (0.315)
**Urine output**	0.007 (0.951)	–0.026 (0.810)
**PO**_**2**_ **(mmHg)/FiO**_**2**_	0.009 (0.934)	0.103 (0.333)
**SNAPII**	–0.294 (0.005*)	–0.233 (0.027*)

TLC: Total leucocyte count, RDW: Red cell distribution width, I/T: Immature/total, RBS: Random blood sugar, CRP: C-reactive protein, ABP: Arterial blood pressure,SNAP: Score of neonatal acute physiology.

*Significant at P <0.05.

### Receiver operating characteristic curve (ROC) analysis

**Fig 2:**
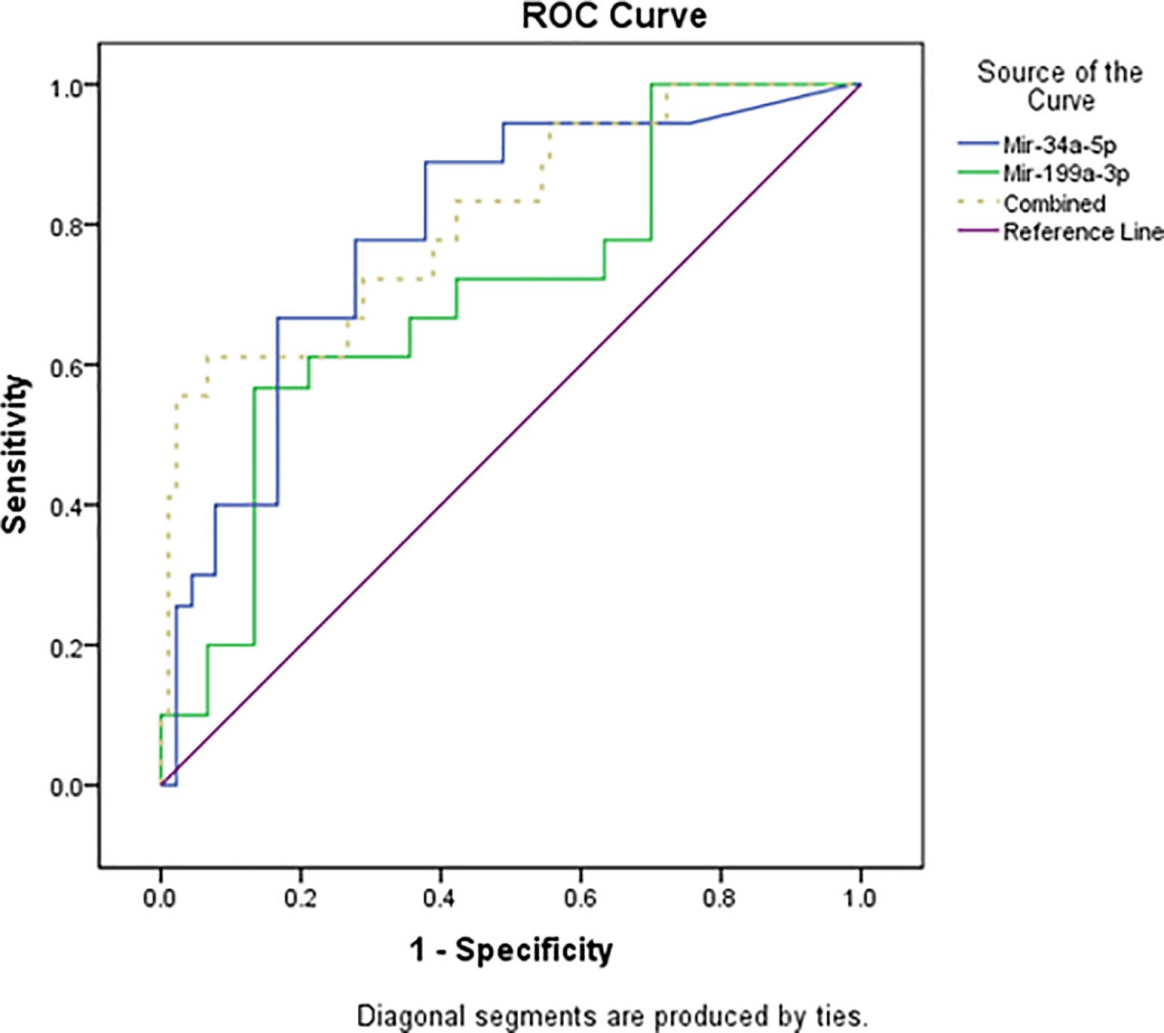
ROC curve analysis of serum miR-34a-5p and miR-199a-3p for differentiating neonates with sepsis from healthy ones.

An ROC curve was created to determine the potential of the serum levels of miR-34a-5p and miR-199a-3p as predictive biomarkers for neonatal sepsis (Fig 2). For miR-34a-5p, the area under the curve (AUC) was 0.792[95%confidence interval (CI): 0.725–0.859] with sensitivity and specificity of 66.7 and 83.3, respectively, and P<0.0001. Moreover, the AUC for miR-199a-3p was 0.701 (95% CI: 0.624–0.778) with sensitivity and specificity of 66.6 and 64.4, respectively, and P<0.0001. The use of a combined model consisting of miR-34a-5p and miR-199a-3p demonstrated an increase of the AUC to 0.812 (95% CI: 0.751–0.874) with sensitivity and specificity of 72.2 and 71.1, respectively, and P <0.0001. However, AUC for IT ratio was 0.864 (95 CI: 0.800–0.928, P<0.0001), which was near to that of combined model with sensitivity = 81.1% and specificity = 96.7 (S1 Fig).

### Multivariate analysis

Predictors that were related to sepsis by a p value of ≤ 0.05by univariate analysis were selected to be used in the multivariable logistic regression model. HR was excluded from the model due to co-linearity with RR. Likewise, GA was excluded as it was correlated with HG and by the same manner HCO3 was related to APGAR score at one minute. Variables as Temperature, PLT, RDW, TLC, Lymph, IT ratio and PH were removed because of either zero or large value of standard error of B coefficient. Along sides with miR-199a-3p and miR-34a-5p, DBP, RR, HG, RBS and APGAR score at one minute were included in the model. After variable selection, Stepwise backward Wald method using p<0.10 for removal was performed (S2 Table).

## Discussion

Neonatal sepsis is a life-threatening condition characterized by an interplay between the host immune system and invading microbial pathogens, resulting in severe systemic inflammation, which leads to multisystem failure [1]. miRNAs are short non coding RNAs that regulate numerous biological processes. Increasing evidence suggests that miRNAs play vital roles in immune regulation in autoimmune and infectious diseases [22,23]. Various biomarkers have been considered to aid the diagnosis and prognosis of sepsis, but with limitations in severe and early cases [2,3]. Recently, much attention has focused on utilizing serum miRNAs as biomarkers for the diagnosis and prognosis of sepsis that are released outside cells into serum and remain stable in circulation, given their ease of detection [24].

It has been reported that miR-34a is a strong tumor suppressor in a variety of cancers [25,26]. The expression of miR-34a-5p has been found to be involved in the regulation of autophagy, cell cycle differentiation, and apoptosis by targeting numerous genes [27,28].

In the present study, we revealed that the serum expression level of miR-34a-5pwas significantly decreased in patients with neonatal sepsis compared with that in healthy neonates.

Our results are in accordance with a previous study that revealed the downregulation of miR-34a in sepsis-induced acute lung injury through targeting fork head box O3 (FoxO3), resulting in the activation of autophagy [29]. Another study by Liu et al. suggested that the inhibition of miR-34a could regulate autophagic activity via the targeting of ATG4B in tubular epithelial cells in acute kidney injury [30]. Additionally, a previous study revealed that miR-34a inhibited autophagy by repressing ATG9A expression in cardiomyocyte hypertrophy [31].

It was noted that autophagy activation, which was proven to regulate innate and acquired immune processes, is a key participant in neonatal sepsis. Numerous microRNAs were documented to play key roles in sepsis through modulating autophagy, such as miR-155, miR-21-3p, miR-300, and miR-23a [32]. We hypothesized that the downregulation of miR-34a-5p in neonatal sepsis could be due to its effect on autophagy, which plays a major role in this condition.

In addition, in a recent study, miR-34a-3p was reported to be notably decreased while signal transducer and activator of transcription 1 (STAT1), cytokine-induced neutrophil chemo attractant 1 (CINC-1), and intercellular adhesion molecule 1(ICAM-1) were increased in sepsis-induced acute lung injury in rats. The aforementioned study proved that sevoflurane reduced the expression of inflammatory markers and improved the viability of lung cells via the upregulation of miR-34a-3p and repression of STAT1 [33].

However, research has shown that miRNA-34a was upregulated in neonatal lung injury patients and mice compared with the level in control groups [34]. Similarly, Cheng et al. confirmed that miR-34a expression was increased in rats with LPS-induced sepsis and U937 [35], which was not in accordance with our results.

The present results showed that miR199a-3p was significantly downregulated in the serum of neonates with sepsis, compared with that in healthy neonates. Our results are in line with a previous study that revealed remarkable downregulation of miR-199a in mice with LPS-induced ALI [36]. In addition, Chen et al. demonstrated that miR-199a-3pexpression was decreased in ALI due to the binding of C-terminal-binding protein 2–histone deacetylase 1–FOXP3 transcriptional complex (CHFTC) to its promoter, resulting in elevation of the expression level of nucleotide-binding oligomerization domain, leucine-rich repeat, and pyrin domain-containing protein 1 (NLRP1), which activates the release of inflammatory markers (IL-1β and IL-18), exacerbating the inflammatory response in ALI [37].

Moreover, it was documented that a significantly reduced level of miR-199a-3p leads to the upregulation of hypoxia inducible factor 1 alpha subunit (HIFA) in HCC [38]. Similarly, Rane et al. proved that miR-199a could regulate the hypoxia-triggered pathway by targeting HIFA [39]. Notably, HIFA plays a key role in the pathogenesis of sepsis [40].

Previous research revealed that miR-199a regulates the IκB kinase-β (IKKb)/nuclear factor-Κb (NF-kB) pathway in ovarian cancer cells [41]. A report has also shown that the IKKb/NF-kB pathway plays a pivotal role in sepsis and is associated with multiple organ failure [42]. Considering these previous studies, we hypothesized that miR-199a-3p might play an important role in the pathogenesis of sepsis.

The current results demonstrated that miR-34a-5p and miR-199a-3p exhibited significant correlations with each of TLC, RDW, RBS, and CRP, as well as SNAPII, indicating their association with the severity of neonatal sepsis. In the line with our results, Chen et al. proved that miR-199a negatively regulated IKK-beta promoting NF-κB signaling activation [41] which in turn participated in endogenous C-reactive protein induction [43]. Moreover, Xie et al. found that miR-34a is positively correlated with numerous inflammatory markers including CRP in rheumatoid arthritis and systemic lupus erythematosus patients [44].

From the above results, we concluded that miR-34a-5p and miR-199a-3p could serve as valuable predictors of neonatal sepsis, with high specificity and sensitivity, and might function as targets for treating this conditions. Furthermore, miR-34a-5p and miR-199a-3p associated significantly with the severity of neonatal sepsis.

In-depth research on a larger population is needed in future work. Further prospective studies should be carried out to investigate the mechanisms of action and the downstream genes of miR-34a-5p and miR-199a-3p during sepsis.

## Supporting information

S1 Fig ROC curve analysis of serum miR-34a-5p, miR-199a-3p and IT ratio for differentiating neonates with sepsis from healthy ones. (TIF)

S1 Table miR-34a-5p and miR-199a-3p fold change in neonates with sepsis. (XLSX)

S2 Table Multivariable logistic regression model. (DOCX)
