# Supplementary figures and images for: Serum miR-34a-5p and miR-199a-3p as new biomarkers of neonatal sepsis
Source: PLoS One. 2022 Jan 6;17(1):e0262339. doi: 10.1371/journal.pone.0262339 (PMC8735601; doi:10.1371/journal.pone.0262339)

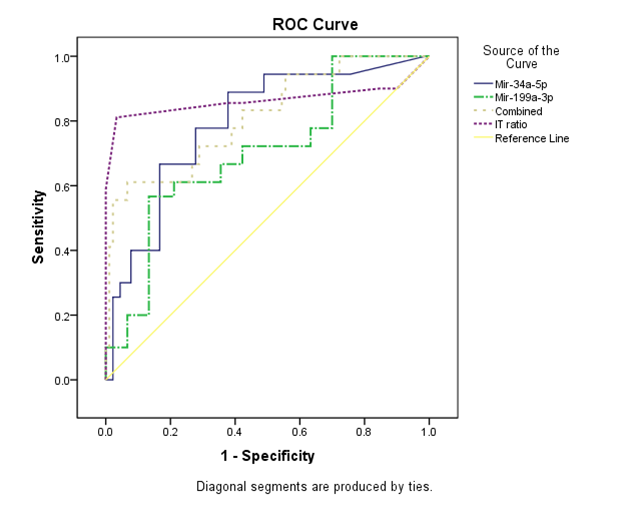

Supplement: S1 Fig — (TIF) [file pone.0262339.s001.tif]
