# Supplementary material for: Serum miR-34a-5p and miR-199a-3p as new biomarkers of neonatal sepsis
Source: PLoS One. 2022 Jan 6;17(1):e0262339. doi: 10.1371/journal.pone.0262339 (PMC8735601; doi:10.1371/journal.pone.0262339)
Supplement: S2 Table — (DOCX) [file pone.0262339.s003.docx]

**Table (4):** multivariate logistic regression analysis

|  | **B** | **S.E for B** | **P-value** | **OR** | **95% C.I. for OR** | |
| --- | --- | --- | --- | --- | --- | --- |
| **Mir34a5p** | **-15.249** | **7.351** | **0.038** | **0.000** | **0.000** | **0.431** |
| **ApgarScore (1Min)** | -2.046 | 0.728 | 0.005 | 0.129 | 0.031 | 0.539 |
| **RR** | 0.268 | 0.072 | 0.000 | 1.308 | 1.137 | 1.505 |
| **DBP** | -0.113 | 0.060 | 0.058 | 0.893 | 0.794 | 1.004 |
| **HG** | -0.795 | 0.227 | 0.000 | 0.451 | 0.289 | 0.705 |
| **RBS** | 0.041 | 0.024 | 0.083 | 1.042 | 0.995 | 1.091 |
| **Constant** | 16.836 | 8.672 | 0.052 | 20503024.934 |  |  |

CI: confidence interval.
